# Determinants of animal disease and nontherapeutic antibiotic use on smallholder livestock farms

**DOI:** 10.3389/fvets.2023.1258214

**Published:** 2024-03-15

**Authors:** Eve Emes, Assèta Kagambèga, Michel Dione

**Affiliations:** ^1^Centre for the Mathematical Modelling of Infectious Diseases, The London School of Hygiene and Tropical Medicine, London, United Kingdom; ^2^Laboratory of Molecular Biology, Epidemiology and Surveillance of Foodborne Bacteria and Viruses, Department of Biochemistry-Microbiology, Doctoral School of Science and Technology, Joseph KI-ZERBO University, Ouagadougou, Burkina Faso; ^3^Animal and Human Health Program, International Livestock Research Institute, Dakar, Senegal

**Keywords:** antimicrobial resistance, livestock, antimicrobial stewardship, One Health, epidemiology, Burkina Faso

## Abstract

**Introduction:**

Reducing nontherapeutic antibiotic use (ABU) in livestock animals has been identified as an important way of curbing the growth of antimicrobial resistance (AMR). However, nontherapeutic ABU may be important for managing animal disease. In order to reduce nontherapeutic ABU, farmers may need to implement other complementary interventions to safeguard animal health and minimize risk. We should therefore investigate if nontherapeutic ABU is associated with better animal health outcomes before advocating to reduce it. We should also investigate non-antibiotic factors which protect animal health and can make nontherapeutic use less necessary, as well as factors which can encourage farmers to improve their antibiotic stewardship.

**Methods:**

The study investigated these questions using data from the AMUSE survey, which is designed to evaluate knowledge, attitudes and practices relating to AMR in smallholder livestock farms. The sample included 320 animal herds from 216 smallholder livestock farms in Burkina Faso, with livestock species including poultry, small ruminants, and cattle. The determinants of the occurrence of animal disease and nontherapeutic ABU were investigated using binary logistic regression.

**Results:**

Results revealed that nontherapeutic ABU was positively associated with animal disease, although the potential reverse causality of this relationship should be investigated further. Going primarily to a public veterinarian for animal health services, and having a higher level of formal education, were negatively associated with the occurrence of disease. Going primarily to a community animal health worker was positively associated with using antibiotics nontherapeutically, whereas going primarily to a public veterinarian was negatively associated with this outcome. Having an animal health professional (of any kind) provide diagnosis and treatment was positively associated with nontherapeutic antibiotic use for goats and sheep.

**Discussion:**

These findings support the expansion of education access and public veterinary services as a way to encourage better antibiotic stewardship while guarding against any animal health risks associated with doing so. They also highlight that animal health professionals other than public veterinarians may prioritize animal health outcomes over antibiotic stewardship goals.

## 1 Introduction

Antimicrobial resistance (AMR), the ability of microbial pathogens to survive in the presence of antimicrobials, is an important and growing danger to human health, environmental health, and food security. The use of antimicrobials (AMU) by humans has resulted in growing rates of AMR (1). The use of antibiotics in livestock animals is one of the biggest forms of AMU, and has been the target of extensive national and international health policy initiatives (2, 3). In particular, international AMR policy targets a reduction in ‘irrational’ AMU in livestock animals, usually referring to nontherapeutic (metaphylactic, prophylactic and growth-promoting) use (4–6).

However, characterizing these uses as always irrational is neither fair nor constructive. While some work has suggested that reducing nontherapeutic antibiotic use in smallholder livestock farms may not worsen animal health or may improve it (7, 8), there is also good evidence of health and productivity benefit from sub-inhibitory doses of antibiotics in livestock animals (9), and previous work from this consortium has pointed to nontherapeutic antibiotic use averting animal disease in smallholder livestock farms (10). In addition to this, the potential growth-promoting effects of antibiotic use in livestock animals may be important for smallholder farmers’ incomes, and for food security generally. This is especially important for countries such as Burkina Faso, which has both a high rate of population growth and a relatively low degree of food security (11, 12). In addition to this, smallholder livestock farmers exist as part of a network of interdependent economic actors which involves marketeers, suppliers, creditors, landlords, pharmaceutical sellers, animal health professionals, and others (13). Simply placing legal restrictions on the use of antibiotics in these farms may not be feasible, and could result in farmers circumventing restrictions by buying substandard or counterfeit antibiotics illegally, which may worsen AMR outcomes.

This gives rise to the problem of how to improve antibiotic stewardship on smallholder livestock farms without potentially endangering animal health or farm productivity, and in a way which farmers are willing to uptake. For this reason, it is important to determine three main things. Firstly, the extent to which nontherapeutic antibiotic use in smallholder livestock farms is important for averting animal disease, assessed here by measuring the association between nontherapeutic AMU and animal disease. Understanding this will help to know if reducing nontherapeutic antibiotic use carries a risk to food security and farmers’ incomes, given that animal disease can negatively affect both of these outcomes.

Secondly, which non-antibiotic measures are associated with animal disease. This gives an insight into factors which could potentially guard against disease, and could therefore be paired with antibiotic use reduction to mitigate risks.

And thirdly, which factors are associated with nontherapeutic antibiotic use. This can give insight into factors which could potentially encourage or facilitate improvements in antibiotic stewardship.

In order to address these three points, the study analyzed data collected using the AMUSE survey (14) among smallholder livestock farmers in peri-urban areas of Ouagadougou. AMUSE is a standardized survey developed by the International Livestock Research Institute to assess knowledge, attitudes and practices (KAP) relating to antibiotic use and resistance in smallholder livestock farms (14). The survey has been used in Burkina Faso (15), Ethiopia (16), Senegal (17, 18), and Uganda (10, 19), and adds to a growing bank of knowledge which can inform agricultural AMR policies at the national and international level. The survey allows results to be compared across contexts, and these survey data have been used to write papers similar to this one focusing on Senegal (17) and Uganda (10).

The study uses binary logistic regression to investigate the determinants of animal disease and nontherapeutic antibiotic use in the smallholder livestock farms surveyed. It aims to use these results to provide insight into the role of nontherapeutic antibiotic use in protecting against disease in this context. It also aims to identify non-antibiotic factors which protect animal health and can reduce the need for nontherapeutic antibiotic use, as well as factors which can encourage farmers to improve their antibiotic stewardship.

## 2 Materials and methods

### 2.1 Study area

Ouagadougou is the most densely populated city in Burkina Faso, West Africa, with 2.4 million inhabitants. The farms surveyed were located in the peri-urban areas on the outskirts of the city.

### 2.2 Study population

**Figure 1:**
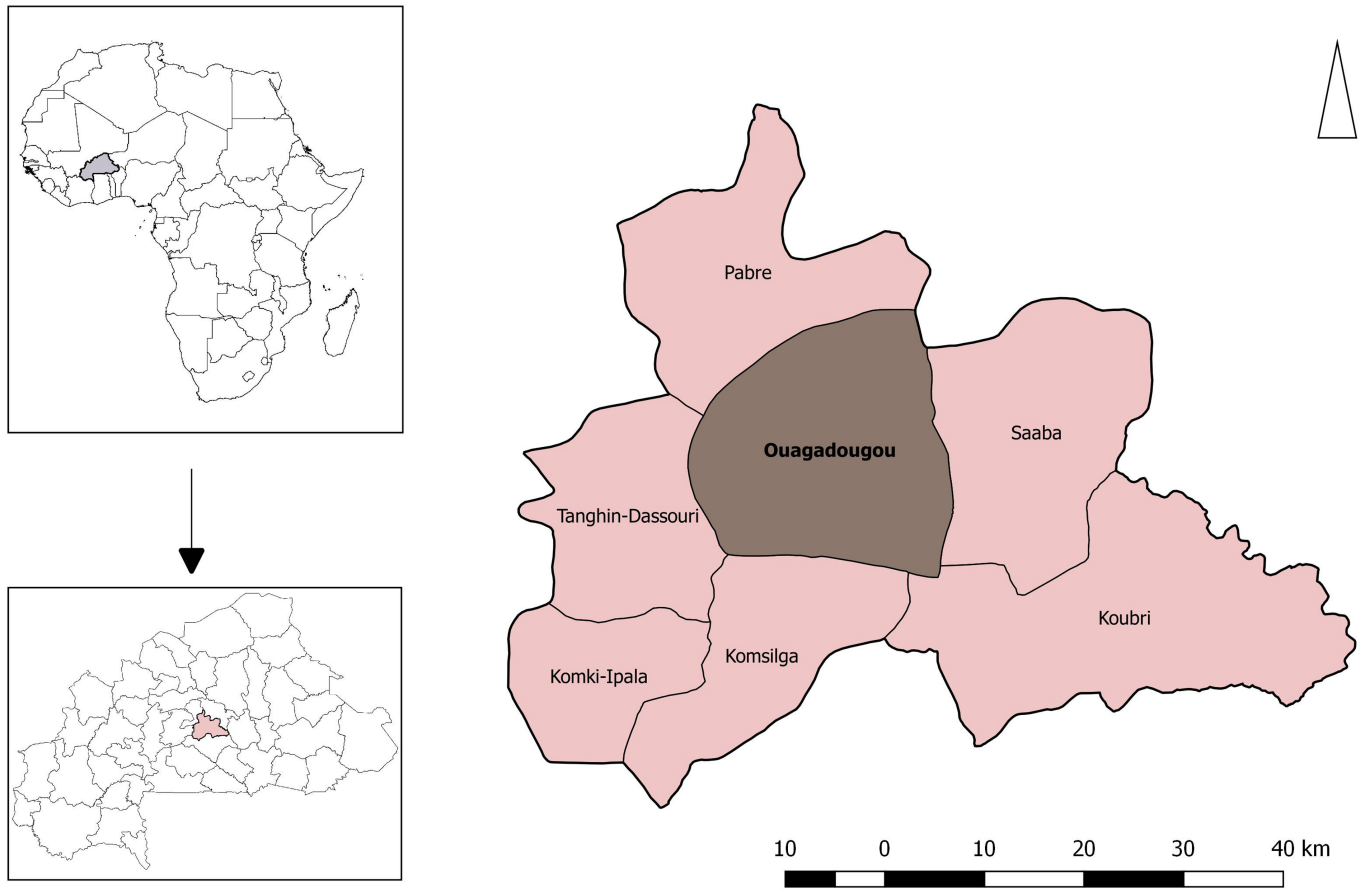
Map of the study area.

A total of 216 farms were surveyed as part of the study. All of the farms were smallholder livestock farms located in the peri-urban areas of Ouagadougou (see Figure 1 for a map of the study area). The livestock species found on the farms included poultry, cattle, and small ruminants (sheep and goats). Some farms had multiple flocks / herds of different species, meaning that from the 216 farms there were 320 flocks / herds included in the sample, and each flock / herd was treated as a separate unit of analysis.

### 2.3 Study design

The study uses data collected using the AMUSE survey tool. It is a retrospective study using data collected by the International Livestock Research Institute (ILRI) as part of a previous study. Survey results were analyzed using both descriptive and inferential statistics (binary logistic regression).

### 2.4 Method of data collection

The study used secondary data from a survey implemented in Burkina Faso between March and July 2020 that evaluated the knowledge, attitudes and practices of smallholder livestock farmers in the peri-urban areas of Ouagadougou, Burkina Faso, with a focus on antibiotics (15). During and after data collection, authors had access to information (including name and gender) which could identify individual participants. Data were collected using Open Data Kit (ODK), a source-based smartphone platform that can be used to create electronic questionnaire forms for real-time data entry. Enumerators interviewed one representative from each farm, either in French or Mooré, depending on the languages spoken by the respondent.

### 2.5 Sampling method and sample size calculation

Farmers were contacted through a directory of farms in Ouagadougou area between March and July 2020. For each farm, the manager (the owner or a designated worker) was contacted and asked to participate in the study. Inclusion criteria were that the farm be a smallholder livestock farm. Farms were excluded if they were large-scale commercial farms or non-livestock farms. The number of farms included in the dataset was determined as part of a previous study (15) for which the data were originally collected, and this study simply made use of that dataset. The sample size of that study was selected to be sufficient to detect differences in the characteristics of farms which did and did not consult a veterinary professional before buying antibiotics, with a risk of error α of 5% and a confidence interval of 95%, assuming that 12.1% of farms did so based on results from a previous study (15, 20).

### 2.6 Data management and statistical analysis

Statistical analyses were performed with RStudio version 2023.03.01 + 446 (21) using R version 4.1.2 (22).

First, survey responses from each farm were compiled into a cross-sectional dataset.^1^ Where farms had multiple flocks and herds of different species, each flock or herd was treated as a separate unit of analysis.

Following cleaning and examining the dataset, two outcomes of interest were selected: the occurrence of disease in the flock or herd in the last 6 months, and the nontherapeutic use of antibiotics (this dataset included data on use for prophylaxis and fattening). Every farm which used antibiotics for fattening also used them for prophylaxis, so we refer to this outcome variable simply as ‘nontherapeutic antibiotic use’ for clarity.

Binary logistic regression was used to investigate the effect of covariates on the likelihood of these outcomes. For both the bivariate and multivariate models, significance was assessed at the 1% (*p* < 0.01), 5% (*p* < 0.05), and 10% (*p* < 0.1) levels, and results were considered significant if they had a *p*-value of *p* < 0.1, as has been the case for other regression-based papers written using the AMUSE survey, as well as the original paper written using this dataset from Burkina Faso (10, 15, 17). All specifications controlled for the number of animals in the flock / herd, given that the occurrence of a single incident of disease or nontherapeutic antibiotic use may increase with the number of animals.

Both binary and numeric variables were used as covariates in the logistic regression specifications. Binary variables included: ‘uses antibiotics prophylactically,’ ‘believes that antibiotics can be used for fattening,’ ‘goes to [particular animal health service provider],’ ‘professional provides diagnosis and treatment,’ and the animal species dummies. Numeric variables included: ‘number of animals in flock/herd’ and ‘formal education level.’ For binary variables, the values displayed in the results tables are the adjusted odds ratios (exponentiated logistic regression coefficients) for the variable being 1 relative to the variable being 0. For numeric variables, the values displayed are adjusted odds ratios (exponentiated logistic regression coefficients) for a unit increase in the variable.

In order to determine which covariates to include in the multivariate models, bivariate models were first run in which each outcome variable was regressed against each covariate individually (controlling for the number of animals in the flock or herd). This was done first for each livestock species (cattle, poultry, sheep and goats) individually, and then for the whole sample (including all flocks and herds surveyed). Whole-sample results included species dummies. Multivariate models were then run for each of the two outcome variables (by livestock species and for the whole sample), including the covariates which were significant in the bivariate models. Separate regressions were run for each livestock species to investigate if the determinants of animal disease and nontherapeutic antibiotic use varied by species.

### 2.7 Ethical approval

The study was approved by the ethical committee of the Ministry of Health, Burkina Faso, with reference number 2020-9-186. Informed (written and signed) consent was obtained from each participant before they were interviewed.

## 3 Results

Bivariate models were first run to select covariates for the multivariate models. The outputs of the univariate models which produced significant results are available in the Appendix.

In the bivariate models, several factors were significantly associated with the occurrence of animal disease. For goats and sheep, and for cattle, prophylactic antibiotic use was positively associated with animal disease. For the sample as a whole, prophylactic antibiotic use was positively associated with animal disease. Having a higher education level and going primarily to a public veterinarian were negatively associated with the occurrence of disease for the sample as a whole. No factors were significantly associated with the occurrence of disease for chickens alone.

Several factors were also significantly associated with the habitual use of antibiotics for nontherapeutic purposes in the bivariate models. For chickens, going primarily to a community animal health worker was positively associated with nontherapeutic AMU. A professional providing diagnosis and treatment, and primarily going to a public veterinarian, were negatively associated with nontherapeutic AMU for chickens.

For goats and sheep, having a professional provide diagnosis and treatment was positively associated with nontherapeutic AMU. For the sample as a whole, going primarily to a community animal health worker was positively associated with nontherapeutic AMU, whereas going primarily to a public veterinarian was negatively associated with nontherapeutic AMU. No factors were significantly associated with nontherapeutic AMU for cattle alone.

Multivariate models were then run for each of the two outcome variables (Tables 1, 2), including the factors which were significant in the bivariate models.

**Table 1:** Determinants of animal disease (adjusted odds ratio).

	Occurrence of disease in last 6 months		
	Goats and Sheep	Cattle	Whole sample
	(1)	(2)	(3)
Uses antibiotics prophylactically	17.559^***^	4.080^*^	2.044^*^
	*p* = 0.001	*p* = 0.072	*p* = 0.062
Primarily goes to a public vet			0.532^*^
			*p* = 0.083
Level of formal education			0.747^**^
			*p* = 0.035
Number of animals in the flock / herd	1.008	1.098^**^	1.000
	*p* = 0.702	*p* = 0.039	*p* = 0.916
Cow dummy			0.069^***^
			*p* = 0.00000
Goats and sheep dummy			0.039^***^
			*p* = 0.000
Constant	0.056^***^	0.084^***^	8.137^***^
	*p* = 0.001	*p* = 0.002	*p* = 0.00003
*N*	59	49	312

^***^Significant at the 1 percent level.

^**^Significant at the 5 percent level.

^*^Significant at the 10 percent level.

**Table 2:** Determinants of habitually using antibiotics for nontherapeutic purposes (adjusted odds ratio).

	Using antibiotics nontherapeutically		
	Chickens and other poultry	Goats and Sheep	Whole sample
	(1)	(2)	(3)
Primarily goes to a community animal health worker	7.265^***^		2.358^**^
	*p* = 0.004		*p* = 0.020
Primarily goes to a public vet	0.432^*^		0.512^*^
	*p* = 0.094		*p* = 0.096
Professional provides diagnosis and treatment	0.438	4.797^**^	
	*p* = 0.114	*p* = 0.020	
Number of animals in the flock / herd	1.001^**^	0.983	1.001^**^
	*p* = 0.013	*p* = 0.497	*p* = 0.042
Cow dummy			0.072^***^
			*p* = 0.00000
Goats and sheep dummy			0.073^***^
			*p* = 0.000
Constant	3.664^***^	0.239^**^	2.940^***^
	*p* = 0.008	*p* = 0.037	*p* = 0.004
*N*	212	59	320

^***^Significant at the 1 percent level.

^**^Significant at the 5 percent level.

^*^Significant at the 10 percent level.

In the multivariate model (Table 1), habitual prophylactic use of antibiotics remained positively associated with the occurrence of disease for goats and sheep, for cattle, and for the sample as a whole. Primarily going to a public veterinarian, and having a higher level of formal education, were both negatively associated with the occurrence of disease for the sample as a whole.

In the multivariate model (Table 2), primarily going to a community animal health worker remained positively associated with using antibiotics nontherapeutically for poultry and for the sample as a whole. By contrast, primarily going to a public veterinarian remained negatively associated with using antibiotics prophylactically for poultry and for the sample as a whole. Having a professional provide diagnosis and treatment remained positively associated with using antibiotics nontherapeutically for goats and sheep, but was no longer significant for poultry.

## 4 Discussion

The study found that habitual prophylactic antibiotic use was consistently positively associated with the occurrence of disease on smallholder livestock farms, whereas having a higher level of formal education and primarily accessing public veterinarians for animal health services were negatively associated with disease.

Primarily going to a community animal health worker for animal health services was positively associated with nontherapeutic antibiotic use, whereas primarily going to a public veterinarian was negatively associated with nontherapeutic antibiotic use. For goats and sheep, having an animal health professional (of any kind) providing diagnosis and treatment was positively associated with nontherapeutic antibiotic use.

It is interesting that habitual nontherapeutic antibiotic use was positively associated with animal disease. This finding is consistent with evidence from farm-level trials in other contexts which suggest that nontherapeutic antibiotic use does not improve, or may actively worsen, animal health outcomes in smallholder livestock farms (8). Other trials suggest that antibiotic stewardship improvements on smallholder poultry farms, when combined with biosecurity interventions and non-antimicrobial food additives, can improve animal health outcomes (7).

However, some studies have identified a positive role for nontherapeutic antibiotics. Earlier studies using the AMUSE survey in Uganda suggested that nontherapeutic antibiotic use guarded against disease in smallholder livestock farms (10). Nontherapeutic antibiotic use may also have benefits for livestock productivity, as evidenced in a study using the AMUSE survey tool in Senegal (17), and there is evidence in the literature that sub-therapeutic doses of antibiotics convey a health and productivity benefit to livestock (9). Our finding of a positive association between animal disease and nontherapeutic antibiotic use may also be subject to reverse causality, as having had more animal disease in the last 6 months may have prompted farmers to adopt more cautious antibiotic use protocols which involve greater nontherapeutic use.

That accessing public veterinary services was negatively associated with disease suggests a positive role in managing animal health. This echoes findings from Uganda that accessing animal health services improved disease outcomes in smallholder livestock farms (10). However, it is worth noting that the same relationship was not observed for other providers of animal health services. That accessing private veterinarians, regardless of qualification status, was not associated with better health outcomes raises questions about the potential for perverse incentives in private antibiotic prescribing. For example, there may be an incentive to sell expensive but inappropriate medicines, a concern raised by stakeholders in the SEFASI consortium’s 2022 workshop in Dakar (23).

It is interesting to note that going primarily to a community animal health worker was positively associated with nontherapeutic antibiotic use, and that having an animal health professional (of any kind) provide diagnosis and treatment was positively associated with nontherapeutic antibiotic use for goats and sheep. This could suggest that animal health professionals do not, by default, prioritize antibiotic stewardship over animal health. This is consistent with results from consultation with poultry industry stakeholders in the UK, who stressed that humanely safeguarding animal health through antibiotics remains an immediate priority for veterinarians (23). The fact that the opposite was true for public veterinarians could mean that they have been more exposed to government goals as part of the ongoing national action plan on AMR in Burkina Faso: these include a drive to involve veterinary medicine in antibiotic stewardship efforts and to change antibiotic prescribing culture (24). In the case of private veterinarians especially, there may also be an incentive to overprescribe to maximize revenue, or to prescribe excessively broad-spectrum antibiotics to minimize the risk of ineffective treatment, a concern raised in previous consortium workshops (23).

This study aimed to identify factors which are associated with animal disease outcomes and nontherapeutic antibiotic use on smallholder livestock farms in Burkina Faso. This addresses the broader goal of identifying potential interventions to facilitate reductions in nontherapeutic antibiotic use while safeguarding against any animal health risks associated with doing so. The results of this study identify expanded public veterinary access as a potential way of achieving both of these goals, and emphasize that not all providers of animal health services are likely to improve antibiotic stewardship outcomes. Improving farmers’ access to education may also help to improve animal health, and therefore to safeguard against health risks associated with reductions in antibiotic use. Studies have emphasized the role of veterinarians’ education in improving AMS outcomes (25), and the value of interventions to improve farmers’ knowledge about AMS (15–17), but there is little literature on the role of formal education in improving AMS outcomes in smallholder livestock farms in this context.

### 4.1 Limitations

Difficulties with the dataset limited the scope of specifications which could be performed. For instance, the small number of farms which used antibiotics intended for humans on animals meant that this could not be included as an outcome. The small number of farms which had taken part in awareness and vaccination campaigns also meant that the effect of this could not be investigated as a covariate. Several livestock species (pigs, rabbits, horses, and donkeys) were represented on only a small number of farms and thus could not be included in the analysis.

Data on the use of drugs in animals only covered the last 4 weeks, meaning that the study could not investigate the effect of drug use frequency on the occurrence of disease due to the potential for reverse causality. The survey used is also a snapshot, giving static information about farm practices and outcomes. A longer-term cohort study could capture changes over time and give insight into the role of covariates in improving farm outcomes over time. Similarly, while this study used observational data, an intervention study could give more specific insight into the most useful ways to improve antibiotic stewardship while safeguarding animal health and farm productivity.

Finally, in any research concning antibiotic stewardship, it must be borne in mind that smallholder farmers exist as part of a complex network of actors which includes lenders, landlords, drug sellers, animal health professionals, marketeers and more (13). Any intervention aiming to improve stewardship outcomes must acknowledge and involve this entire network.

## 5 Conclusion

Using a survey of smallholder livestock farms in Burkina Faso, this study found that there was a greater likelihood of animal diseasrms where habitual prophylactic antibiotic use was observed. This contradicts the authors’ original hypothesis that prophylactic antibiotic use may protect against animal disease, although the relationship observed may be subject to reverse causality. It also found that there was a lower likelihood of animal disease when farmers had a higher level of formal education, or went primarily to public veterinarians for animal health services (as opposed to other animal health service providers).

The study also found that primarily going to a community animal health worker was positively associated with using antibiotics nontherapeutically, whereas primarily going to a public veterinarian was negatively associated with that outcome. Having an animal health professional (of any kind) provide diagnosis and treatment was also positively associated with nontherapeutic antibiotic use in goats and sheep.

These findings highlight the potential of expansion of education access and public veterinary services as a way to encourage better antibiotic stewardship while safeguarding against any animal health risks associated with reducing nontherapeutic antibiotic use. They also highlight that some types of animal health professional may prioritize animal health outcomes over antibiotic stewardship goals.

Future research should involve farm-level trials and qualitative studies to examine the relationship between nontherapeutic antibiotic use and animal disease in more detail, to explore the extent to which different animal health service providers face incentives to overprescribe, and to test the effect of expanded public veterinary access on antibiotic stewardship and animal health outcomes.

Finally, smallholder farmers form part of a complex network of actors, and this whole network must be considered when designing and implementing antibiotic stewardship policies.

## Data availability statement

The data analyzed in this study is subject to the following licenses/restrictions: anonymised data can be made available upon request. Requests to access these datasets should be directed to eve.emes@lshtm.ac.uk.

## Ethics statement

The studies involving humans were approved by the Ethical Committee of the Ministry of Health, Burkina Faso, with reference number 2020-9-186. Informed (written and signed) consent was obtained from each participant before they were interviewed. Consequently, all participants gave their consent to participate in the study. The studies were conducted in accordance with the local legislation and institutional requirements. Written informed consent for participation was not required from the participants or the participants’ legal guardians/next of kin in accordance with the national legislation and institutional requirements. Written informed consent was obtained from the individual(s) for the publication of any potentially identifiable images or data included in this article.

## Author contributions

EE: Conceptualization, Data curation, Formal analysis, Methodology, Software, Validation, Visualization, Writing – original draft, Writing – review & editing. AK: Conceptualization, Data curation, Funding acquisition, Investigation, Methodology, Writing – review & editing. MD: Writing – review & editing, Conceptualization, Data curation, Funding acquisition, Investigation, Methodology, Project administration, Resources, Supervision.

## Footnotes

^1^A cross-sectional dataset is one which includes observations from multiple subjects at a single point or period in time.
