# Supplementary material for: Determinants of animal disease and nontherapeutic antibiotic use on smallholder livestock farms
Source: Front Vet Sci. 2024 Mar 15;10:1258214. doi: 10.3389/fvets.2023.1258214 (PMC10978738; doi:10.3389/fvets.2023.1258214)
Supplement: Supplementary file 1 [file Data_Sheet_1.docx]

**Supplementary Material**

**Supplementary Table 1:**


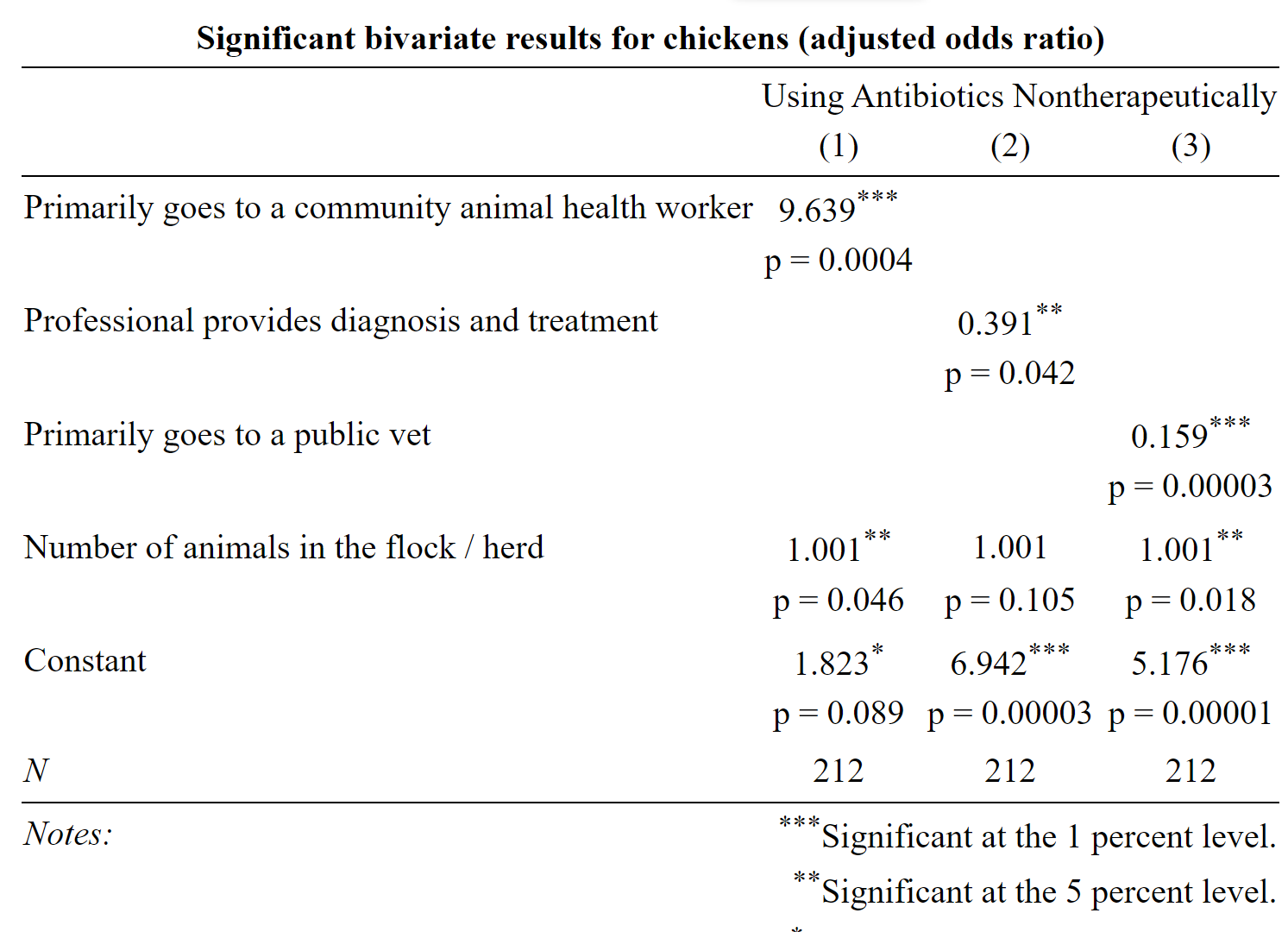


**Supplementary Table 2:**

**
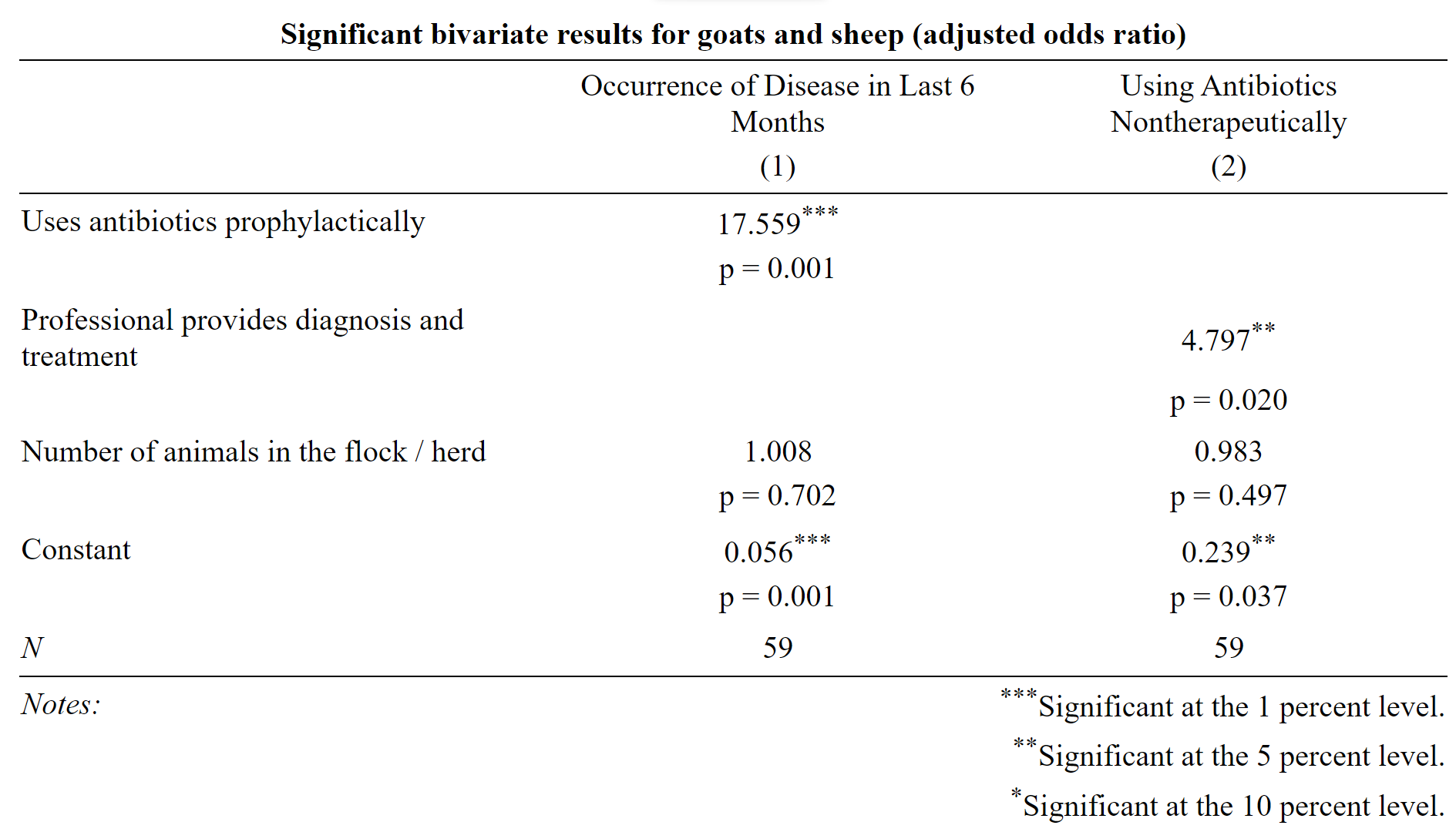
**

**Supplementary Table 3:**

**
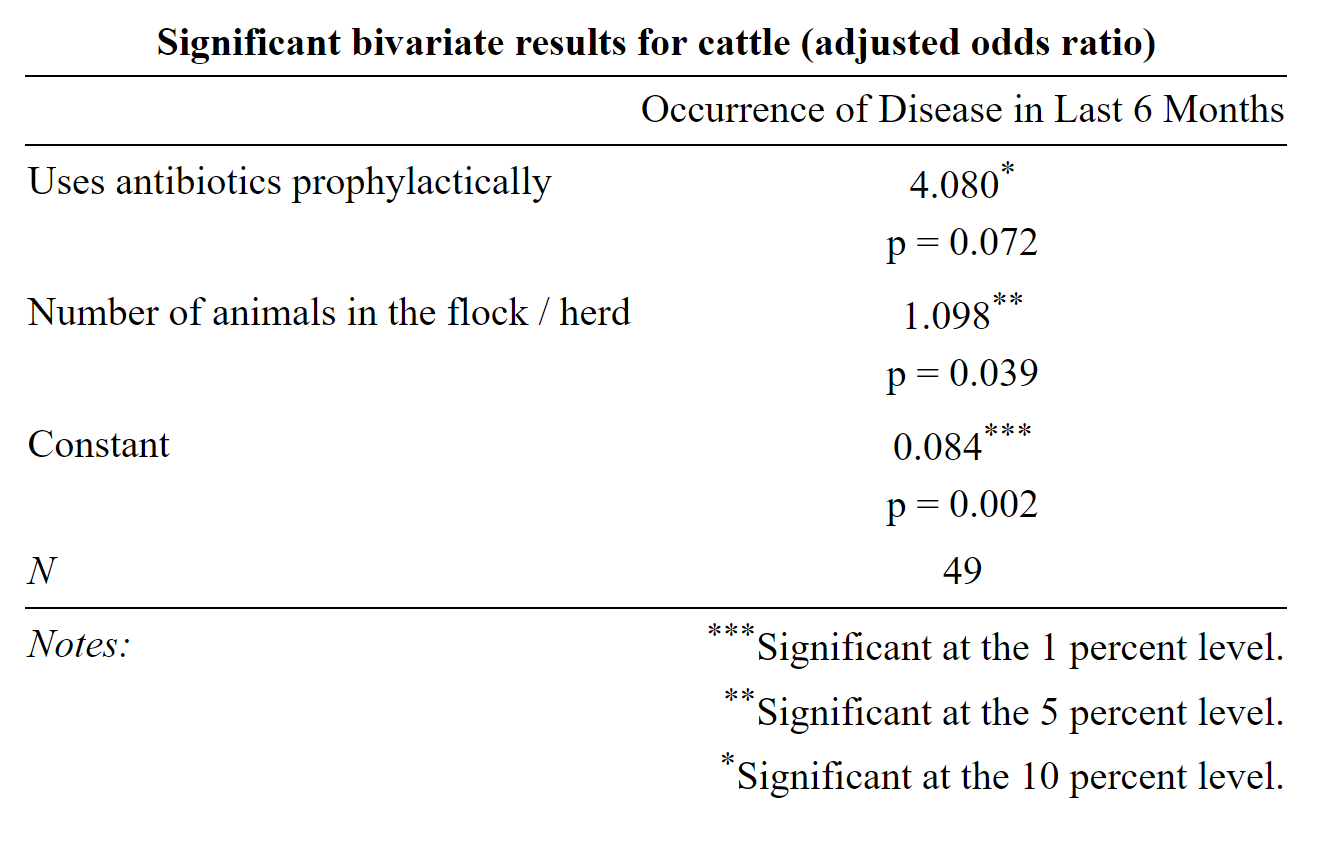
**

**Supplementary Table 4:**

**
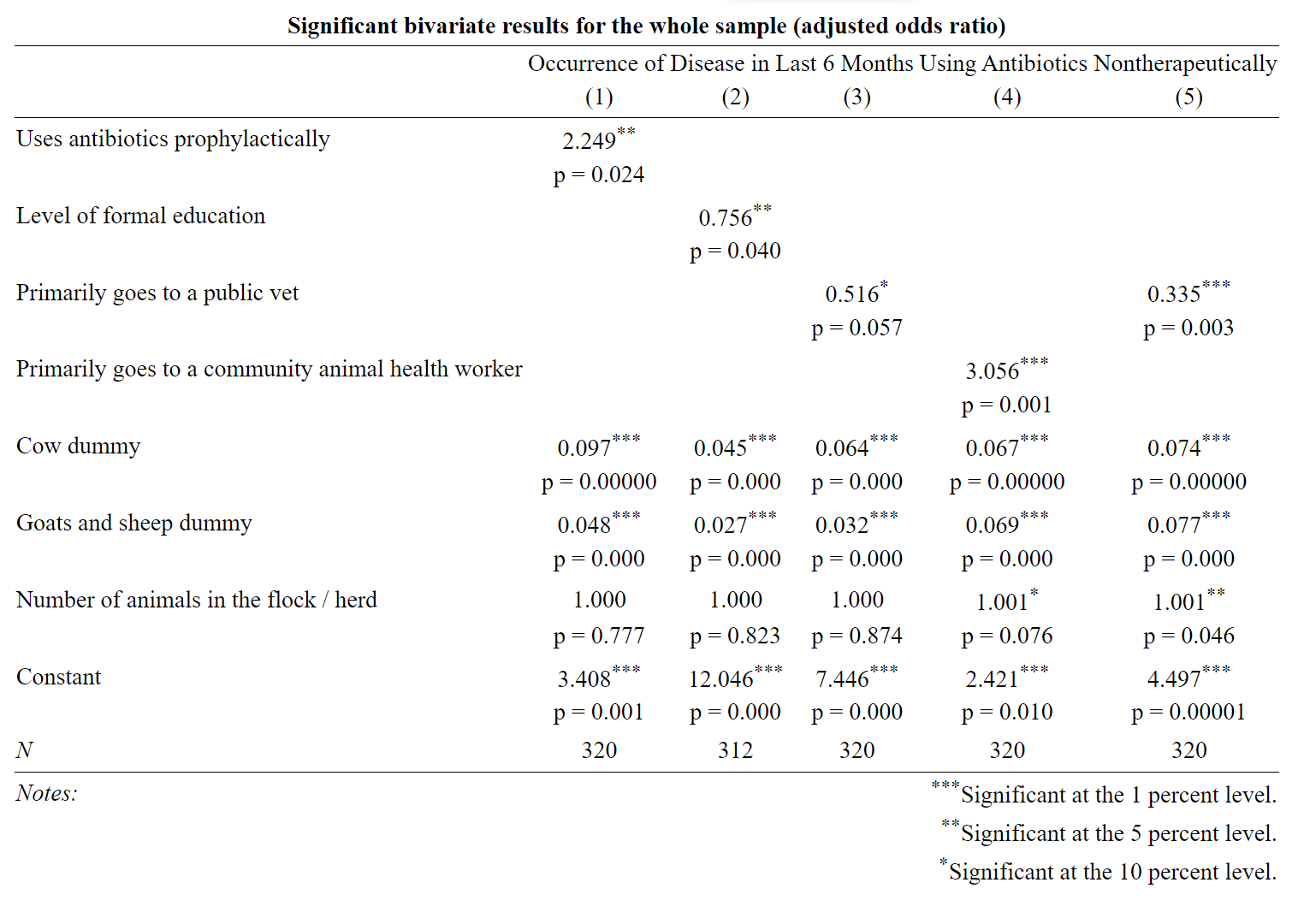
**
